# Splanchnic Oxygen Saturation Is Not Associated With Early Growth in Very Preterm Infants

**DOI:** 10.1159/neo/adsag023

**Published:** 2026-10-10

**Authors:** Baukje M. Dotinga, Bineta E. Lahr, Sijmen A. Reijneveld, Arend F. Bos, Roy E. Stewart, Elisabeth M.W. Kooi

**Affiliations:** a Department of Pediatrics, Division of Neonatology, Beatrix Children’s Hospital University Medical Center Groningen, University of Groningen, Groningen, The Netherlands; b Department of Health Sciences, University Medical Center Groningen, University of Groningen, Groningen, The Netherlands

**Keywords:** Preterm infants, Growth, Intestinal circulation, Regional oxygen saturation, Near-infrared spectroscopy

## Abstract

**Introduction:**

Preterm birth is associated with poorer growth, possibly due to postnatal conditions that disrupt nutritional status. Previous studies found an association between low regional splanchnic oxygen saturation (r_s_SO_2_) and feeding intolerance (FI). We hypothesized that low r_s_SO_2_ is associated with poorer growth because of FI and associated impaired digestion and absorption.

**Methods:**

We included infants with gestational age (GA) <32 weeks and/or birth weight <1.2 kilograms (kg). We excluded infants with chromosomal or congenital abnormalities, gastrointestinal diseases and those who died. We collected daily 2-hour mean r_s_SO_2_ measurements, using near-infrared spectroscopy, until the age of 7 days, and daily weight until the age of 21 days. We calculated the weekly relative growth rate (RGR) as the change in weight (g) divided by weight at the start of the interval (kg) and by the number of days. We used latent growth curve models to assess the association between r_s_SO_2_ and RGR.

**Results:**

We included 214 infants with a mean GA of 29.6 weeks and a mean birth weight of 1.4 kg. We did not find an association between r_s_SO_2_ in the first 7 days and RGR in the first 21 days of age. Relative growth rate in the first 2 weeks after birth was higher in lower GA and those born small for gestational age (SGA). Time to reach full enteral feeding was longer in lower GA and in SGA, with GA showing the stronger association.

**Conclusion:**

Longitudinal r_s_SO_2_ is not associated with early growth outcomes of very preterm infants.

## Introduction

Preterm birth is associated with poorer growth in early childhood, likely resulting from unfavorable extra-uterine conditions and perinatal complications that disrupt the nutritional support for infant growth [1]. Nutritional status is challenged in preterm infants. Energy expenditure may be higher due to thermoregulation, respiratory distress, infection, and other illnesses, while stores of key nutrients are lower [1, 2]. In addition, intestinal immaturity may lead to feeding intolerance (FI) and reduced enteral intake [2, 3]. Finally, intestinal oxygenation required for digestion and absorption may be compromised by immature mechanisms and conditions affecting oxygen supply and demand, including anemia, hypotension, and hypothermia [4].

Monitoring regional splanchnic oxygen saturation (r_s_SO_2_), using near-infrared spectroscopy (NIRS), has gained interest [5]. Although its use in routine care is limited, potential applications include identifying gastrointestinal complications [5]. Early introduction and faster advancement of enteral feeding may promote intestinal development and reduce the need for parenteral nutrition and central lines yet potentially increase the risk of necrotizing enterocolitis (NEC), characterized by ischemic bowel injury and a high risk of long-term sequelae [6]. Several studies evaluated hemodynamic responses to enteral feeding and its relationship with gastrointestinal outcomes. Most demonstrated a postprandial increase in r_s_SO_2_ after bolus feeding, particularly with advancing gestational age (GA) and feeding volumes, while findings for regional cerebral oxygen saturation (r_c_SO_2_) were less consistent [7–13]. Additionally, several studies showed that lower r_s_SO_2_ at baseline and during enteral feeding is associated with symptoms of FI [14, 15].

We hypothesized that low r_s_SO_2_ is associated with poorer growth, either directly through cellular oxygen deficiency leading to poorer digestion and absorption or indirectly through FI. Therefore, the primary aim of this pilot study was to assess the association between r_s_SO_2_ and early growth outcomes and secondarily time to reach full enteral feeding (FEF).

## Methods

### Patients

We conducted a single-center observational study using the sample of a prospective cohort trial. All infants with GA <32 weeks and/or birth weight <1.2 kg admitted to our level IV neonatal intensive care unit (NICU) between January 2016 and December 2017 were eligible. Infants were excluded in case of chromosomal or congenital abnormalities, gastrointestinal diseases including volvulus, spontaneous intestinal perforation, and NEC (Bell’s stage ≥2), congenital heart defects, or death before discharge from the NICU [16]. The study was approved by the medical ethical committee of the University Medical Center Groningen (Approval No.: METc 2017/024).

According to our feeding protocol, enteral feeding was initiated with 15–20 mL/kg/day within 24 hours after birth and increased by 15–20 mL/kg/day, as tolerated. Volumes were not increased, or decreased, in case of abdominal distension, emesis, bilious gastric residuals, or gastric residuals >5 mL. Infants received mother’s own milk when available, or preterm formula (PF, 80 kcal/100 mL). Pasteurized donor human milk was not routinely available during the study period and was therefore not used. Mother’s own milk was fortified with breast milk fortifier after 1 week if an enteral intake of 80 mL/kg/day had been achieved. Mother’s own milk and PF were fortified with protein fortifier after 15 days if FEF had been achieved. Full enteral feeding was defined as ≥150 mL/kg/day for 3 consecutive days. Enteral feeds were given as boluses via a nasogastric tube every 2 hours if birth weight was <1.25 kg and every 3 hours if birth weight was ≥1.25 kg. Parenteral nutrition was initiated from birth and consisted of glucose, amino acids, and lipids. Enteral feeding contributed to total fluid intake from an intake of ≥15 mL/kg/day. Parenteral nutrition was gradually reduced as enteral feeding volumes increased and discontinued after FEF was achieved.

### NIRS Measurements

R_s_SO_2_ and r_c_SO_2_ monitoring were part of routine care and performed 2–3 times daily for at least 2 hours during the first week. Measurements were obtained using an INVOS 5100C near-infrared spectrometer (Medtronic, Dublin, Ireland) and neonatal SomaSensors (Medtronic) on Mepitel^®^ sheets (Mölnlycke Health Care, Göteborg, Sweden) to protect the skin. The splanchnic sensor was placed infra-umbilical on the central abdomen and the cerebral sensor on either frontoparietal side of the head. Sensor position was checked by the attending nurse and verified daily by a researcher. If an umbilical catheter or infant size prevented adequate sensor placement, r_s_SO_2_ measurements were not performed. Peripheral oxygen saturation (SpO_2_) was recorded simultaneously using Nellcor^®^ pulse oximeters (Medtronic). According to our local protocol during the study period, target SpO_2_ was 90%–92%, with alarm settings set at 86% and 93%. R_s_SO_2_, r_c_SO_2_, and SpO_2_ were stored in an offline, pseudonymized database every 5 seconds. We calculated daily mean r_s_SO_2_ and r_c_SO_2_ using 2-hour periods containing at least 80% of data. If >2 hours of data were available, we selected the period closest to the documented time of sensor placement verification. Artifacts indicating sensor misplacement (unexplained sudden nonphysiological changes or prolonged absence of physiological variability) were removed manually. The splanchnic-cerebral oxygenation ratio (SCOR = r_s_SO_2_/r_c_SO_2_) was calculated to assess the balance between intestinal and cerebral oxygenation.

### Other Data Collection

Weight data from birth to 21 days were obtained from medical records. Infants were weighed daily during the first postnatal week and/or until regaining birth weight and at least 3 times weekly thereafter. Infants were classified as small for gestational age (SGA) if their birth weight was <10th percentile using Dutch reference values [17]. Relative growth rate (RGR, g/kg/day) was calculated as weight change (g) over each 7-day interval divided by body weight (kg) at the start of the interval. Time to reach FEF was recorded as days from birth, even if FEF was achieved after 21 days.

Maternal and pregnancy characteristics included parity, multiple gestation, and mode of delivery. Perinatal characteristics included GA, head circumference at birth, sex, and 5-minute Apgar score. Illness severity was assessed using the Score for Neonatal Acute Physiology with Perinatal Extension-II (SNAPPE-II) [18]. Neonatal characteristics included echocardiography-confirmed hemodynamically significant ductus arteriosus (hsPDA), sepsis (clinical symptoms plus a positive blood culture and/or C-reactive protein >20 mg/L), intraventricular hemorrhage (IVH) grade III/IV, mechanical ventilation, inotropic support, and blood transfusions. Daily measurements during the first week after birth included hemoglobin level (mmol/L; within ±12 hours of each r_s_SO_2_ measurement), type of feeding, volume of feeding (mL/kg/day), SpO_2_, r_s_SO_2_, r_c_SO_2_, and SCOR.

### Statistical Analyses

Assuming data were missing at random, missing r_s_SO_2_ and r_c_SO_2_ values were imputed using predictive mean matching to allow valid inference. The imputation model included GA, SNAPPE-II, head circumference at birth, weight from birth to day 21, type and volume of feeding, previous and following SpO_2_ and hemoglobin levels. We generated 25 imputed datasets and pooled estimates for all analyses.

We used data from the first week to build separate linear growth models for r_s_SO_2_ and SCOR, estimating an intercept (initial level) and slope (change over time) for each parameter. Gestational age and SGA were included in the models. Correlations between RGR at days 8, 15, and 21 were also estimated. Associations between longitudinal r_s_SO_2_ and time to reach FEF were assessed using the same linear growth model, adjusting for GA and SGA. Univariable linear regression analyses assessed associations between daily r_s_SO_2_ values and hsPDA, IVH grade III/IV, and sepsis.

As this was an exploratory study, no formal power analyses or adjustments for multiple comparisons were performed. *p* values <0.05 were considered statistically significant.

Statistical analyses were performed with IBM Statistical Package for the Social Sciences (SPSS, IBM Corp., Armonk, NY, USA) version 26.0, Statistical Analysis System (SAS, SAS Institute, Cary, NC, USA) version 9.4, and Mplus (Muthén and Muthén, Los Angeles, CA, USA) version 8.9.

## Results

Of 278 infants eligible for inclusion, 64 were excluded due to chromosomal abnormalities (*n* = 5), congenital heart defects (*n* = 2), gastrointestinal disease, including NEC (*n* = 23), death before NICU discharge (*n* = 14), or a combination of these criteria (*n* = 20). The final study population comprised 214 infants. Baseline characteristics are shown in Table 1. Mean GA was 29.6 ± 1.9 weeks, and mean birth weight was 1.4 ± 0.4 kg. Fifty infants (23%) were born SGA. Common comorbidities included hsPDA in 45 infants (21%), sepsis in 23 (11%), and IVH grade III/IV in 13 (6%). Mechanical ventilation was required in 114 infants (53%), inotropic support in 14 (7%), and blood transfusions in 78 (37%). Time to reach FEF was 13 ± 6 days (Table 1). The daily hemoglobin, feeding, growth, and oxygenation measurements are summarized in Table 2.

**Table 1.** Clinical Characteristics of Participating Infants and Their Mothers

Maternal and pregnancy characteristics	*n* = 214
	Number (%) or mean±SD
Multiparae	78 (36)
Multiple gestation	
Singleton	152 (71)
Twin	56 (26)
Triplet	6 (3)
Caesarean section	86 (40)
Perinatal and neonatal characteristics	
GA (weeks)	29.6±1.9
Birth weight (kg)	1.4±0.4
Head circumference at birth (cm)	27.2±2.4
Female	96 (45)
5-Min Apgar score	8±1
SNAPPE-II score	13±16
SGA	50 (23)
hsPDA	45 (21)
Sepsis	23 (11)
IVH grade III/IV	13 (6)
Mechanical ventilation	114 (53)
Need for inotropic support	14 (7)
Need for blood transfusion	78 (37)
Time to reach FEF (days)	13±6

Data are presented as mean ± standard deviation or number (percentage).

GA, gestational age; SNAPPE-II, Score for Neonatal Acute Physiology with Perinatal Extension-II; SGA, small for gestational age; hsPDA, hemodynamically significant patent ductus arteriosus based on echocardiographic findings; IVH, intraventricular hemorrhage; FEF, full enteral feeding.

**Table 2.** Daily Hemoglobin, Feeding, Growth, and Oxygenation Measurements

Clinical variables	Day 1	Day 2	Day 3	Day 4	Day 5	Day 6	Day 7	Day 14	Day 21
Hemoglobin (mmol/L)	10 0.4±1.6	9.7±1.8	10.0±1.6	9.6±1.8	9.3±1.7	9.3±1.7	8.9±2.5	–	–
Feeding									
Volume (mL/kg)	2±3	14±7	28±12	43±17	57±22	70±28	82±33	–	–
Type (% MM)	60±50	40±40	50±40	70±40	80±30	90±30	90±30	–	–
Growth									
Weight (kg)	1.4±0.4	1.4±0.4	1.3±0.3	1.2±0.3	1.2±0.3	1.2±0.3	1.2±0.3	1.3±0.3	1.4±0.4
RGR (g/kg/day)	–	–	–	–	–	–	−3±11	16±9	20±10
SpO_2_ (%)	94±4	94±4	94±3	95±3	95±3	95±3	95±3	–	–
r_s_SO_2_ (%)	53±17	51±18	44±18	40±18	37±18	39±18	39±17	–	–
r_c_SO_2_ (%)	81±9	80±8	79±10	78±9	76±10	76±9	74±9	​	​
SCOR	0.7±0.2	0.6±0.2	0.6±0.2	0.5±0.2	0.5±0.3	0.5±0.3	0.5±0.2	​	​

Data are presented are mean ± standard deviation.

MM, mother’s own milk; RGR, relative growth rate; SpO_2_, peripheral oxygen saturation; r_s_SO_2_, regional splanchnic oxygen saturation; r_c_SO_2_, regional cerebral oxygen saturation; SCOR, splanchnic-cerebral oxygenation ratio.

### Regional Splanchnic Oxygen Saturation and Early Growth

We found no significant association between longitudinal r_s_SO_2_ and early growth (Table 3). Infants reached their lowest postnatal weight at 4 ± 2 days (range 1–11). To assess confounding, GA and SGA were included in the models separately and together. The associations remained nonsignificant after adjustment. Univariable analyses showed that hsPDA was associated with lower r_s_SO_2_ on postnatal days 2, 3, and 7, whereas IVH grade III/IV was associated with lower r_s_SO_2_ on postnatal day 3. Sepsis was not associated with r_s_SO_2_ (online suppl. Table 1; for all online suppl. material, see https://doi.org/10.1159/neo/adsag023). Early growth was associated with lower GA and SGA in the first 2 weeks (i.e., days 1–7 and days 8–14) and with SGA in the third week (i.e., day 15–21). SCOR intercept was associated with early growth (days 1–7) in the unadjusted model (β = −0.322, *p* = 0.010) but not after adjustment for GA and SGA (online suppl. Table 2). We did not find enteral feeding, expressed as the average volume of mother’s own milk during the studied period, or any other potential confounders to add to the models with statistical significance (data not shown).

**Table 3.** Association Between Regional Splanchnic Oxygen Saturation and Relative Growth Rate

Variables	Model 1		Model 2		Model 3		Model 4	
	β	*p* value	β	*p* value	β	*p* value	β	*p* value
Growth days 1–7								
r_s_SO_2_ intercept	−0.195	0.957	−0.019	0.993	−0.164	0.913	0.045	0.979
r_s_SO_2_ slope	0.202	0.977	0.333	0.930	0.114	0.962	0.268	0.926
GA	–	–	−0.357	<0.001*	–	–	−0.387	<0.001*
SGA	–	–	–	–	0.402	<0.001*	0.426	<0.001*
Growth days 8–14								
r_s_SO_2_ intercept	−0.139	0.388	−0.041	0.896	−0.015	0.585	−0.012	0.957
r_s_SO_2_ slope	−0.008	0.978	0.078	0.853	−0.020	0.952	0.050	0.876
GA	–	–	−0.188	0.039*	–	–	−0.200	0.022*
SGA	–	–	–	–	0.165	0.023*	0.178	0.014*
Growth days 15–21								
r_s_SO_2_ intercept	0.115	0.873	0.179	0.849	0.135	0.806	0.198	0.849
r_s_SO_2_ slope	0.183	0.912	0.244	0.905	0.159	0.882	0.208	0.918
GA	–	–	−0.100	0.353	–	–	−0.115	0.287
SGA	–	–	–	–	0.163	0.033*	0.169	0.026*

Data are presented as regression coefficients (β) with corresponding *p* values. Model 1 included regional splanchnic oxygen saturation (r_s_SO_2_) intercept and r_s_SO_2_ slope; model 2 included r_s_SO_2_ intercept, r_s_SO_2_ slope, and gestational age in total days (GA); model 3 included r_s_SO_2_ intercept, r_s_SO_2_ slope, and small for gestational age (SGA); model 4 included r_s_SO_2_ intercept, r_s_SO_2_ slope, GA, and SGA.

*p < 0.05.

### Regional Splanchnic Oxygen Saturation and Time to Reach FEF

Similarly, we found no significant association between longitudinal r_s_SO_2_ and time to reach FEF (Table 4). Gestational age and SGA were included in the models for FEF as covariates, with higher GA showing the strongest association with a shorter time to reach FEF and non-SGA status showing a weaker secondary effect.

**Table 4.** Association Between Regional Splanchnic Oxygen Saturation and Time to Reach Full Enteral Feeding

Variables	Model 1		Model 2		Model 3		Model 4	
	β	*p* value	β	*p* value	β	*p* value	β	*p* value
r_s_SO_2_ intercept	−2.27	0.746	2.60	0.713	−1.24	0.861	4.92	0.493
r_s_SO_2_ slope	−0.52	0.775	−0.72	0.683	−0.63	0.725	−0.92	0.596
GA	–	–	−3.89	<0.001*	–	–	−4.50	<0.001*
SGA	–	–	–	–	0.38	0.086	0.58	0.011*

Data are presented as regression coefficients (β) with corresponding *p* values. Model 1 included regional splanchnic oxygen saturation (r_s_SO_2_) intercept and r_s_SO_2_ slope; model 2 included r_s_SO_2_ intercept, r_s_SO_2_ slope, and gestational age in total days (GA); model 3 included r_s_SO_2_ intercept, r_s_SO_2_ slope, and small for gestational age (SGA); model 4 included r_s_SO_2_ intercept, r_s_SO_2_ slope, GA, and SGA.

*p < 0.05.

## Discussion

In this study, longitudinal r_s_SO_2_ in the first week after birth was not associated with early growth in preterm infants or time to reach FEF. Conversely, lower GA and SGA status were associated with higher RGR and longer time to reach FEF.

To the best of our knowledge, we are the first to explore the association between r_s_SO_2_ and early growth. Previous studies reported an association between lower r_s_SO_2_ and symptoms of FI, e.g., abdominal distension, gastric residuals, and bloody stools [14, 19]. Cortez et al. [14] reported consistently lower r_s_SO_2_ during the first week in infants with GA <30 weeks who later developed FI. Similarly, Corvaglia et al. [19] studied the response to the first enteral feeding in infants with GA <33 weeks and reported that lower r_s_SO_2_ at baseline, during, and after feeding was associated with later development of FI. In contrast, Dani et al. [15] demonstrated that a single r_s_SO_2_ measurement between 24 and 72 hours after birth was not associated with time to reach FEF. We confirmed this in a larger sample using longitudinal r_s_SO_2_ during the first week. Therefore, lower r_s_SO_2_ does not translate to a longer time to FEF or poorer early growth, contrary to our hypothesis.

Several factors may explain the lack of an association between r_s_SO_2_ and early growth. First, although adequate oxygenation is essential for intestinal functioning, early nutritional uptake may be determined mostly by gastrointestinal maturation. This is supported by the strong association of early growth with GA and SGA. Additionally, during the transition from parenteral to enteral nutrition, growth in preterm infants is largely maintained by parenteral nutrition, which may have masked any relationship between splanchnic oxygenation and growth outcomes. Consistent with this, SCOR was not associated with early growth after adjustment for GA and SGA, suggesting that relative differences in intestinal and cerebral oxygenation do not explain variation in early growth. Second, NIRS-derived r_s_SO_2_ may not reliably reflect intestinal oxygenation as measurements can be influenced by other tissues, intestinal contents, and bowel movements [8, 20]. Nevertheless, NIRS correlated with blood flow velocity in the superior mesenteric artery and venous saturation [8, 20]. Finally, as r_s_SO_2_ mainly reflects venous saturation of the splanchnic vascular bed, lower values may indicate greater oxygen extraction rather than insufficient oxygenation for digestion and nutrient absorption. Investigating r_s_SO_2_ variability rather than mean values may provide additional insights into intestinal function and growth [21]. This variability may reflect physiological processes such as peristalsis, with regional fluctuations in oxygenation. In conditions like NEC, peristalsis is reduced, and inflammation may alter intestinal blood flow, leading to decreased variability despite relatively high r_s_SO_2_ values [22].

RGR was higher with lower GA and in SGA status, whereas FEF was achieved faster at higher GA and non-SGA status. Smaller or less mature infants may show higher relative rates due to lower body weight, despite remaining clinically more vulnerable [23]. This is consistent with normative preterm growth data, which show higher weight-gain velocities at lower GA that decline with increasing maturity [24]. Greater morbidity, higher energy expenditure, and immature gut and metabolic function may limit efficient nutrient uptake and feeding progression [1–3]. These factors could contribute to slower progression to FEF in the most immature infants, even when relative growth rates appear higher [23].

Strengths of our study include its large sample and longitudinal design, allowing us to evaluate the change in r_s_SO_2_ during the first week. However, several limitations should be acknowledged. First, missing values required imputation based on related variables. Second, feeding times were not recorded, although enteral feeding may influence r_s_SO_2_. Most studies included infants with higher GA and larger feeding volumes than ours [7–13]. In comparable study populations, no postprandial increase in r_s_SO_2_ has been observed [12]. However, Du et al. [25] reported that the postprandial response may differ depending on feeding tolerance, with an increase in r_s_SO_2_ observed in feeding-tolerant infants and a decrease in infants with FI. Although enteral feeds were administered at 2–3-hour intervals and each 2-hour period used to calculate daily mean r_s_SO_2_ likely included a bolus feeding, differences in postprandial responses between infants may have influenced our measurements [25]. Furthermore, common neonatal morbidities may influence r_s_SO_2_. Lower r_s_SO_2_ was associated with hsPDA and IVH grade III/IV on selected postnatal days, although these associations were inconsistent over time. Finally, the 21-day observation period may be relatively short for assessing growth as preterm infants typically regain birth weight at 7–10 days [26]. As our center is a level IV NICU, infants were generally transferred to secondary care hospitals after the initial intensive care period, limiting longitudinal growth data beyond this period. In our cohort, however, lowest postnatal weight occurred at a mean of 4 days and no later than 11 days, indicating that the 15–21-day window reflected true growth for all infants.

In conclusion, we found no association between r_s_SO_2_ and early growth or time to reach FEF. This suggests that r_s_SO_2_ alone may be insufficient to reflect growth-related intestinal physiology, particularly when GA and SGA are considered. Future studies could assess r_s_SO_2_, including its variability, and early growth by analyzing hemodynamic responses to enteral feeding using simultaneous r_s_SO_2_ and superior mesenteric artery blood flow measurements. Integrated assessment of intestinal oxygenation and perfusion may help to characterize early growth in preterm infants.

## Acknowledgments

We acknowledge the patients and their parents for participating in the cohort study. Furthermore, we acknowledge the support of the nurses and staff of the University Medical Center of Groningen neonatal intensive care unit with the data collection.

## Statement of Ethics

The Medical Ethics Committee of the University of Groningen confirmed that this observational study was not subject to the Dutch Medical Research Involving Human Subjects Act (WMO) (Approval No.: METc 2017/024). Therefore, formal ethical approval and the requirement for informed consent were waived.

## Conflict of Interest Statement

Prof. Dr. Arend F. Bos was a member of the journal’s Editorial Board at the time of submission. The other authors have no conflict of interest to declare.

## Funding Sources

B.E.L. received financial support from the Junior Scientific Masterclass, Graduate School of Medical Sciences, University of Groningen (MD-PhD 23-17). The funder had no role in the design, data collection, data analysis, and reporting of this study.

## Author Contributions

B.M.D.: conceptualization, data curation, formal analysis, investigation, methodology, resources, and writing – original draft; B.E.L.: conceptualization, data curation, formal analysis, investigation, methodology, resources, and writing – original draft; S.A.R.: conceptualization, methodology, supervision, and writing – review and editing; A.F.B.: conceptualization, methodology, supervision, and writing – review and editing; R.E.S.: formal analysis, software, validation, and writing – review and editing; E.M.W.K.: conceptualization, data curation, funding acquisition, methodology, project administration, resources, supervision, validation, and writing – review and editing. All authors approved the final manuscript and agreed to be accountable for all aspects of the work.

## Data Availability

The data sets generated and/or analyzed during the current study are not publicly available due to ethical and privacy restrictions but are available from the corresponding author upon reasonable request and with appropriate approval.
